# Properties and Hydration Mechanism of Soda Residue-Activated Ground Granulated Blast Furnace Slag Cementitious Materials

**DOI:** 10.3390/ma14112883

**Published:** 2021-05-27

**Authors:** Yonghui Lin, Dongqiang Xu, Xianhui Zhao

**Affiliations:** 1 School of Civil and Transportation Engineering, Hebei University of Technology, Tianjin 300401, China; 2002021@hebut.edu.cn; 2 Department of Development and Domestic Cooperation, Hebei Normal University for Nationalities, Chengde 067000, China; 3 School of Civil Engineering, Hebei University of Engineering, Handan 056038, China; zhaoxianhui@hebeu.edu.cn

**Keywords:** soda residue, ground granulated blast furnace slag, alkali activation, microstructure, hydration products, chloride binding, cement paste

## Abstract

Soda residue (SR), an industrial solid waste, pollutes the environment due to its high alkalinity and chloride ion content. SR can be used as an alkali activator of ground granulated blast furnace slag (GGBFS). This study investigated the effects of four types of SR-activated GGBFS cementitious materials (pastes) with different mass ratios of SR to GGBFS (8:92, 16:84, 24:76, 34:68) on the physical properties, mechanical strength, and chloride binding capacity. The hydration mechanism of the pastes was also studied. Results showed that with the increasing addition of SR, the density of the pastes decreased, and more white aggregates of SR appeared causing the increase of water absorption and porosity of the pastes. The pastes with 16% SR addition had the maximum compressive strength (34.1 MPa, 28 d), so the optimum proportion of SR addition in the pastes was 16%. With the increases of SR addition, the amount of chloride element in the initial pastes increases. When the proportion of SR addition is 8%, the mass percentage of free chloride ion in the pastes at 28 d is 0.13%. The main hydration products of the pastes were C–S–H gels, ettringite, and Friedel’s salt, and the amount of ettringite varied with the amount of SR addition and curing time.

## 1. Introduction

Soda ash (Na_2_CO_3_, also called sodium carbonate) is an important chemical raw material widely used in the chemical industry. The Solvay process, developed into its modern form by Ernest Solvay during the 1860s, generates more than 60% of the soda ash in China [1]. It produces Na_2_CO_3_ from brine (as a source of NaCl) and from limestone (as a source of CaCO_3_) [2,3]. The overall process is shown in Equation (1). The Solvay process produces solid waste, mainly including soluble matter (CaCl_2_, NaCl) and suspended matter (CaCO_3_, CaSO_4_, Ca(OH)_2_, SiO_2_, and Al_2_O_3_) [4].

(1) $$2\mathrm{NaCl}+{\mathrm{CaCO}}_{3}\to {\mathrm{Na}}_{2}{\mathrm{CO}}_{3}+{\mathrm{CaCl}}_{2}$$

China produced at least 4.72 million tons of soda residue (SR) in 2018 [5], which was mainly discharged into landfills or the sea. With the increase of soda ash production capacity, the discharge of SR is increasing. The storage capacity of landfills is becoming smaller and smaller, and the maintenance cost of landfills higher and higher. Besides, if SR cannot be utilized effectively and in a timely way, it will occupy a large amount of land (In China, more than 15 km^2^ of land was occupied) [6]. In addition, the SR leaching pollutes the soil and groundwater [7]. The air-dried SR produces blowing dust due to the wind [6]. These all represent hazards to the ecological environment [8]. 

Several studies have been carried out to utilize SR for neutralizing acidic soil [3], stabilizing heavy metals [9] and soft clay [10,11], producing cement [12], synthesizing geopolymers [13,14], and preparing ceramics [15]. However, the effective utilization rate of SR is less than 5% [16], thus it is worthwhile to investigate the comprehensive utilization of SR so as to benefit the environment and the economy.

In recent years, the cement industry is facing challenges such as increasing energy supply costs, requirements to reduce CO_2_ emissions [17], and lack of a sufficient supply of raw materials [18]. Simultaneously, the utilization of alkaline waste (especially those coming from the chemical industry, such as carbide slag, desulphurization gypsum, and SR) as alkali activators has become an important area of research in many laboratories. Alkaline waste can be used to synthesize inexpensive and eco-friendly cement-like construction materials. SR mainly contains CaCO_3_, CaSO_4_, Ca(OH)_2_, CaCl_2_, and NaCl, and its pH value is 10 to 12. Therefore, it is likely to become a useful material in civil engineering. Wang et al. [7] added SR to cement raw material to synthesize cement clinker, and the compressive strength of the produced cement was 53.1 MPa at 28 d, but its freezing-thawing resistance was weak. Zhao et al. [13,16] prepared the polymer with SR, fly ash and different alkaline activators (NaOH, Na_2_SiO_3_, Ca (OH)_2_, one or more of them), and analyzed the effects of SR particles and curing temperature on the strength of the geopolymer. According to their study, the 28 d compressive strength of NaOH-activated SR (20%) and fly ash (80%) based geopolymer was only 33% of the 150 d, mainly due to the slow hydration reaction of fly ash. Yang et al. [19] studied the performance of concrete using SR as a mineral admixture and found that the original SR and the washed SR caused steel corrosion due to high chloride ion content; only the SR with 0.30% chloride content could be used as mineral admixtures. Therefore, to utilize SR in the civil engineering field, the solidification of chloride ion is essential.

Ground granulated blast furnace slag (GGBFS) is a by-product material of iron, and its main chemical components are CaO, SiO_2_, and Al_2_O_3_ [20,21]. GGBFS, usually classified as a latent hydraulic material, acts only as activation materials [18,20]. Numerous studies have been testified that GGBFS can partly replace ordinary Portland cement (OPC) for saving energy and reducing carbon dioxide (CO_2_) emission [22,23]. It is worth noting that cement-based mortars made of GGBFS have a weak carbonation resistance [24,25], so a minimum wet curing time of seven days is needed [24]. Besides, GGBFS can combine with alkaline activators (CaO, Ca(OH)_2_, NaOH, KOH, Na_2_SiO_3_·nH_2_O, and K_2_SiO_3_·nH_2_O, one or more of them) to form alkali-activated GGBFS (AAS) to fully replace cement [5,23]. GGBFS has been considered an environmentally friendly material. AAS has excellent properties (early and high mechanical strengths [26], low hydration heat [27], durability [12], resistance to chemical attack [28] and freeze-thaw cycles [26]).

It is commonly believed that GGBFS can consume Ca(OH)_2_ through the hydration of cement. Studies also show that the addition of CaO or Ca(OH)_2_ can accelerate the hydration of GGBFS [29,30]. High pH value is the main factor to promote the hydration of GGBFS. The main hydration products of Ca(OH)_2_-activated GGBFS cementitious materials are CSH (xCaO-ySiO_2_-nH_2_O) and CAH (xCaO-yAl_2_O_3_-nH_2_O) [29,31]. Additionally, the hydration products of Ca(OH)_2_ activated GGBFS binder (mass ratio of Ca(OH)_2_ to GGBFS is 6.5:93.5) vary with the curing time. With the increase of curing time, Ca(OH)_2_ is consumed in hydration, and carbonation takes place in the binder to form calcium carbonates [32]. As shown in Equation (2) [33] and Equation (3) [34], when chloride or sulfate is present in the environment, GGBFS has an excellent binding ability with sulfate and chloride to form ettringite (AFt, 3CaO⋅Al_2_O_3_⋅3CaSO_4_⋅32H_2_O) and Friedel’s salt (Fs, 3CaO⋅Al_2_O_3_⋅CaCl_2_⋅10H_2_O) [10,35,36,37], respectively:

(2) $$3\mathrm{CaO}+{\mathrm{Al}}_{2}O_{3}+3{\mathrm{CaSO}}_{4}+32H_{2}O\to 3\mathrm{CaO}\cdot {\mathrm{Al}}_{2}O_{3}\cdot 3{\mathrm{CaSO}}_{4}\cdot 32H_{2}O$$

(3) $$3\mathrm{CaO}+{\mathrm{Al}}_{2}O_{3}+{\mathrm{CaCl}}_{2}+10H_{2}O\to 3\mathrm{CaO}\cdot {\mathrm{Al}}_{2}O_{3}\cdot {\mathrm{CaCl}}_{2}\cdot 10H_{2}O$$

The high chloride and sulfate binding capacity of AAS may be due to the high alumina content in slag, resulting in the formation of Fs and Aft, respectively. Numerous studies [35,38,39,40] have been carried out on the properties and products of cement-based materials under chloride, sulfate, or chloride-sulfate corrosion. What is more, related researches mainly focus on the erosion of exosmotic ions, while few researches were carried out on the mechanism of product transformation in cement mixed with chloride and sulfate. Zang et al. [41] investigated the interaction between chloride ion (3.5%) and sulfate ion (0.5%) in cement binders. They found chloride ion could restrain the generation of ettringite, resulting in the conversion from AFt to Fs and increase of chemically bound chloride ion.

This study attempts to utilize SR and GGBFS to prepare SR-activated GGBFS cementitious materials (pastes). Four kinds of pastes with different mass ratios of SR to GGBFS (8:92, 16:84, 24:76, 34:68) were prepared. Density, water absorption, porosity, compressive strength, and flexural strength of the pastes were measured to determine the proper proportion of SR in the pastes. X-ray diffraction (XRD), fourier transform infrared spectroscopy (FTIR), and scanning electron microscopy and x-ray energy spectrometer (SEM-EDS) were applied to investigate the micro-characteristics of the pastes. Finally, the hydration mechanism of the pastes was studied. The utilization of waste (SR) and byproducts (GGBFS) can help reduce waste and relieve environmental pressure.

## 2. Materials and Methods

### 2.1. Materials

Raw materials for the preparation of the pastes are SR and GGBFS. Physical and chemical properties of SR and GGBFS determine properties and hydration products of the pastes. In this experiment, SR was industrial waste from Tangshan Sanyou Chemical Industries Company Limited (Tangshan, Hebei Province, China). GGBFS was obtained from Tangshan Xinrong Slag Powder Company Limited (Tangshan, Hebei Province, China) [5]. Chemical compositions and physical properties of SR and GGBFS are listed in Table 1.

**Figure 1:**
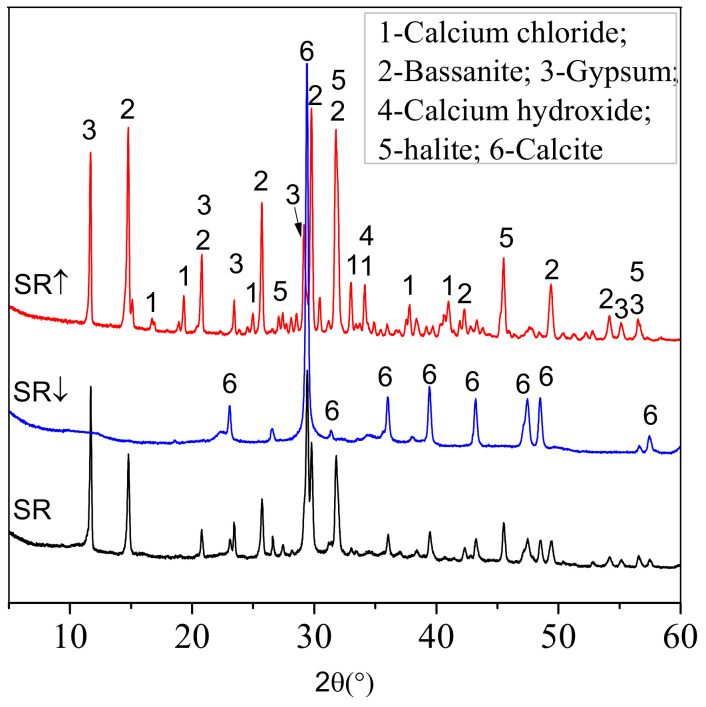
XRD patents of supernatant (SR↑), precipitation (SR↓), and SR.

In this experiment, the raw SR was firstly dried (105 ± 5 °C) and then ground to pass through a 200 mesh sieve. Then it was immersed in water (mass ratio of SR to water is 1:100) and stirred for 3 min (285 ± 10 rpm). Next, the mixture was filtered with filter paper to obtain supernatant and precipitate. After that, they were dried respectively to a constant weight, and tested by XRD (Rigaku, Tokyo, Japan). The phases of supernatant, precipitate, and SR are shown in Figure 1. The main phases of upernatant are calcium chloride, bassanite, gypsum, calcium hydroxide, halite and calcite. The main phase of precipitate is calcite. However, no characteristic peaks of calcium hydroxide and calcium chloride can be found in the XRD patterns of SR.

**Figure 2:**
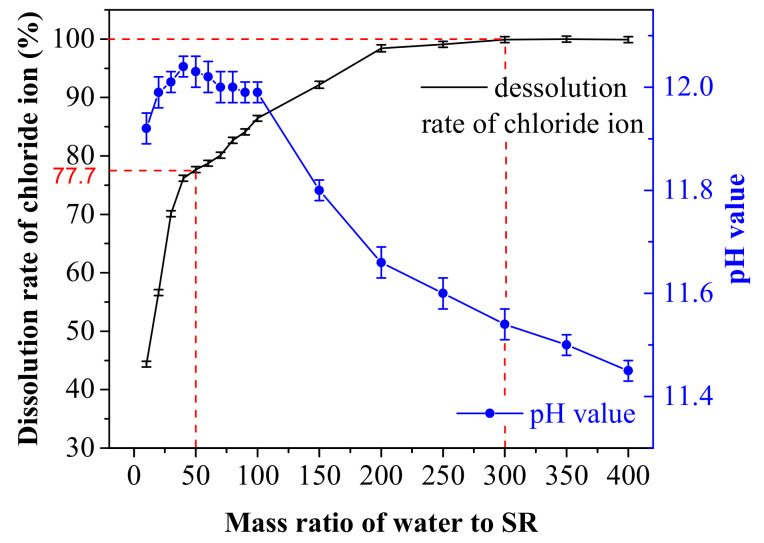
Dissolution rate of chloride (**black**) and pH value (**blue**) with different mass ratio of water to SR.

SR was mixed with water in mass ratios of 1:10 to 1:400. The pH value and chloride ion concentration of its supernatant were tested. As Figure 2 shows, the average dissolution rate of chloride and pH value vary with the mass ratio of water to SR. As mass ratio of water to SR increases, the dissolution rate of chloride ion rises. When mass ratio of water to SR is 50:1, the dissolution rate of chloride ion is 77.7%. When the mass ratio of water to SR reaches 300:1, dissolution rate of chloride ion is 100%, indicating that a large amount of water is needed to dissolve all the chloride ion in the SR. The pH value firstly increases and then decreases. The maximum pH value of supernatant is 12.04 when water to SR mass ratio is 40:1. The solution of SR is still in a high alkaline environment (pH value > 11.4) when the mass ratio of water to SR is 400:1. 

**Figure 3:**
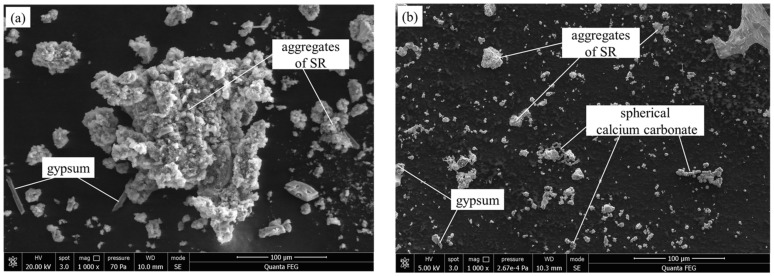
SEM images of SR: (**a**) the raw SR and (**b**) the stirred SR.

Scanning electron microscopy (SEM) images of the raw SR and the stirred SR are shown in Figure 3. As Figure 3a shows, the raw SR has a rough and honeycomb-like structure, with many large pores, and the particles are cemented together by aragonite and gypsum. As Figure 3b shows, aggregates with a diameter of about 10 μm can be seen after stirring for 3 min (285 ± 10 rpm), and spherical calcium carbonate can be observed. This indicates that mechanical force can break the raw SR into small aggregates, which may fill in the pores between the particles.

### 2.2. Preparation of Specimens

In this study, four kinds of SR-activated GGBFS pastes, SG1, SG2, SG3, and SG4, were synthesized, in which the mass ratios of SR to GGBFS were 8:92, 16:84, 24:76, and 32:68, respectively. These ratios were selected based on a preliminary test [5]. The water to powders (SR and GGBFS) mass ratio was 0.5. SR and GGBFS powders were firstly well mixed for 3 min, and then water was added and mixed for another 3 min. After that, the homogenized paste was molded into prismatic molds (40 mm × 40 mm × 160 mm) with two layers based on GB/T 17671-1999 (ISO) standard (China) [42]. Each layer was vibrated for 1 min to remove entrained air. The prepared specimens with molds were then placed in sealed plastic bags, cured in a chamber at 25 ± 3 °C and 95 ± 5% RH (relative humidity). After 1 d of curing, the demoulded specimens continued to be sealed and cured in the chamber until testing at 3, 7 and 28 d. After the compressive strength test, the fragments of hardened pastes were ground and immersed in ethanol for 8 h, and then vacuum dried at 50 °C for 24 h to interrupt the hydration.

### 2.3. Testing and Characterization

#### 2.3.1. Chemical Compositions

The mass percentage of CaCO_3_ in SR was measured by the gravimetric method. First, SR was dried (105 ± 5 °C) to a constant weight and weighed (*A*) in a flask (2 L, weight *B*). Then, excess HCl (0.1 mol/L, weighed *C*) was added slowly to the volumetric flask until all the SR was dissolved. Next, the flask and the solution in it were weighed (weight *D*). The mass percentage of CaCO_3_ in SR (*Cc*) was calculated by Equation (4):

(4) $$Cc=\frac{\left(A+B+C-D\right)\times 100}{A\times 44}\times 100\%$$

where 44 and 100 are molar mass (g/mol) of CO_2_ and CaCO_3_, respectively. The mass percentage of chloride element in SR was measured by PXSJ-216F ion meter (Shanghai Yidian Science Instrument Company Limited, Shanghai, China) [43]. Na, Mg, Al, Si, S, and Ca elements in SR were measured by X-ray fluorescence (XRF)(Rigaku, Tokyo, Japan). Na, Mg, Al, Si, S, K, Ca, Ti, and Fe elements in GGBFS were measured by XRF. Finally, compositions of SR and GGBFS were calculated by element conservation method.

#### 2.3.2. Density, Water Absorption and Porosity

According to the ASTM C20-2000(2005) standard [44], the physical properties (density, water absorption, and porosity) of the pastes were tested. First, the 28 d-cured specimens were dried under 105 ± 5 °C for 24 h, and then cooled in a dryer to 25 ± 1 °C, and weighed (weight *A*). Second, the dried specimens were immersed in water (21 ± 1 °C) for 48 h, the surface of the specimens was wiped with a damp cloth, and then the specimens were weighed (weight *B*). Third, the soaked specimens were put into boiling water, and boiled for 5 h, and then naturally cooled to 25 ± 1 °C, wiped, weighed (weight *C*). Last, the boiled specimens in water were weighed (weight *D*). Apparent density (*ρ*), water absorption (*W*), and porosity (*P*) were calculated by Equations (5)–(7), respectively, where *ρ*_H2O_ is the density of water:

(5) $$\rho =\frac{A}{A-D}\times \rho_{H_{2}O}$$

(6) $$W=\frac{B-A}{A}\times 100\%$$

(7) $$P=\frac{C-A}{C-D}\times 100\%$$

#### 2.3.3. Compressive and Flexural Strength 

Compressive and flexural Strength of specimens were measured on a model YAW-300 machine (Shanghai Suns Machinery Manufacturing Co., Ltd., Shanghai, China) with 50 and 2400 N/s loading rate, respectively [5]. Average values of compressive and flexural strength were measured from twelve and six specimens, respectively.

#### 2.3.4. Phase Evolution

XRD, SEM-EDS, and FTIR were used to analyze the phases and the hydration products of SR and the pastes. The XRD data were collected on a Rigaku D/Max-2500 X-ray diffractometer (Akishima, Tokyo, Japan) with CuKα radiation generated at 40 kV and 150 mA. The measurements were made from 5° to 60° at 4°/min. The microstructure of SR was carried out by SEM (Quanta FEG450, Hillsboro, OR, USA) at 20 kV, 1000×. Morphology observation and chemical analysis of the pastes were carried out by SEM-EDS (Quanta JEOL JSM-7610FPlus, Rigaku, Tokyo, Japan). SEM images were taken at 1~5 kV (depending on the electrical conductivity of the material), 10,000×, and EDS at a 20 kV accelerating voltage. Characteristics of the bonds were analyzed by FTIR (Tensor 27, Bruker, Karlsruhe, Germany) with the wavenumber ranging from 400 to 4000 cm^−1^. 

#### 2.3.5. Dissolution of Chloride Ion

The dried sample (20 g) and deionized water (200 mL) were mixed in a triangular flask, vibrated for 2 min, and soaked for 24 h. After that, 20 mL chloride ion protector (0.1 mol/L KNO_3_) were added to 20 mL filtrate taken from the mixture. The pH value [45] and the chloride ion concentration (C1) was tested immediately with PXSJ-216F ion meter [43]. When the total chloride ion content (*C*) was measured, 200 mL deionized water in the above experiment was replaced with HNO_3_ (2 mol/L). The chloride binding rate (*P_s_*) was calculated by Equation (8):

(8) $$P_{s}=\frac{C1}{C}\times 100\%$$

## 3. Results and Discussion

### 3.1. Physical Properties of the Pastes

**Figure 4:**
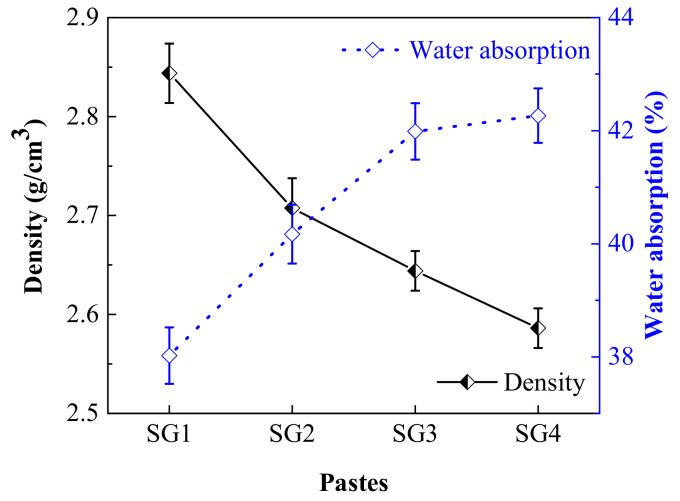
Density (**black**) and water absorption (**blue**) of the pastes at 28 d.

The measured results of density and water absorption of the pastes at 28 d are shown in Figure 4. Density of the pastes decreases with the increasing proportions of SR addition. This result agrees with the study of Zhao et al. [13]. The decrease of the density is mainly due to the increasing addition of SR, as the density of raw SR and GGBFS are 2.252 and 2.873 g/cm^3^, respectively. The water absorption of the pastes rises with the increasing SR addition. Previous research [5] indicated that SR had strong water absorption. Additionally, Figure 3 shows that many pores exist in SR. These are some of the reasons why water absorption of the pastes increases with the increase of SR addition. 

**Figure 5:**
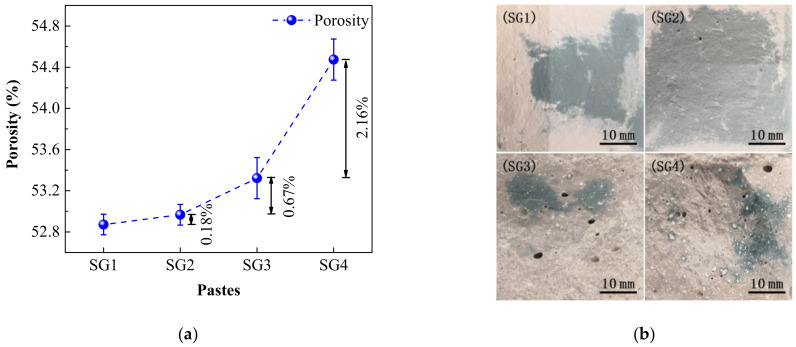
Porosity (**a**) and fracture surfaces (**b**) of the pastes at 28 d.

Figure 5 shows the porosity of the pastes and fracture surfaces (40 mm × 40 mm) after flexural tests at 28 d. It can be seen that the porosity of the pastes increases with the increasing proportion of SR addition (Figure 5a). Compared with SG1, the porosity of SG2 increases only by 0.18% when the SR addition increases by 8%. However, with the increase of SR addition, the porosity increases significantly. Compared with SG3, the porosity of SG4 increases by 2.16%. It can also be seen from the fracture surfaces of SG1, SG2, SG3, and SG4 (Figure 5b) that with the increasing addition of SR, larger pores and white aggregates of SR appear in SG3 and SG4. It indicates that small proportion of SR addition makes the pastes much denser as CaCO_3_ in SR can fill the pores, whereas large proportion of SR can easily lead to aggregates and uneven distribution in the pastes, causing the increase in porosity and water absorption. What is more, pores bigger than 200 nm in cementitious materials are much harm pores and have been considered as one of the main factors influencing strength [23]. Therefore, high porosity and visible pores in SG3 and SG4 may weaken the mechanical properties of the pastes.

### 3.2. Mechanical Properties of the Pastes

**Figure 6:**
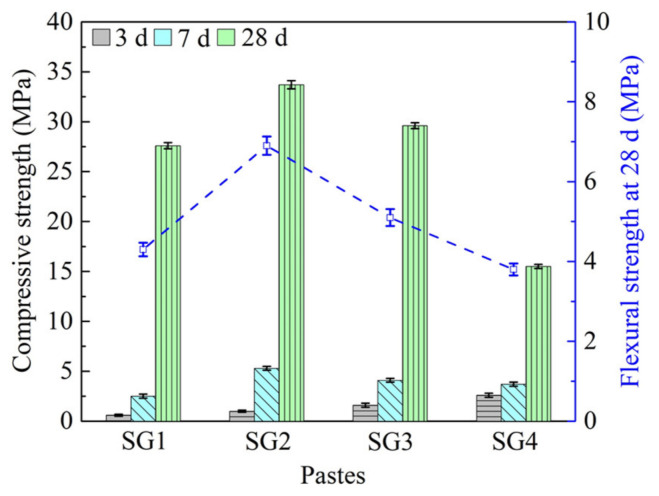
Compressive strength (**left**) and flexural strength (**right**) of the pastes.

On the basis of the previous research [5], the test data values of compressive and flexural strength increased. The average measured results of compressive strength (3, 7, and 28 d) and flexural strength (28 d) of the pastes are given in Figure 6. The compressive strength of the pastes increases with SR proportion rising from 8 to 32% at 3 d. However, with the increase of SR addition, the compressive strength (7 d and 28 d) and the flexural strength (28 d) firstly increases and then decreases. The maximum compressive strength (34.1 MPa) and flexural strength (6.9 MPa) occur when the weight ratio of SR to GGBFS is 16:84, at 28 d. Therefore, the optimum proportion of SR addition is 16% in the pastes. The decline of mechanical strength may due to high porosity and water absorption of the pastes (the proportion of SR addition: 24–32%), and aggregate and uneven distribution of SR in the pastes (Figure 5). This indicates that more than 16% SR addition will lead to low strength of the pastes. It can also be seen from Figure 6, compressive strength of all the pastes increases with the increase of curing time. A study by Sanjuán et al. [24] showed that the compressive strength of cement with different proportions of GGFBS addition at 2 d can reach 25–65% (also depending on the kinds of cements) of that at 28 d. Additionally, no matter what the ratio of SR to GGBFS is, the early compressive strength (3d, 7d) of the pastes is far lower than that at 28 d. The slow speed of the Ca^2+^ ions dissolution from GGBFS [46], and CaCO_3_ in SR are the main reasons for the low early compressive strength [5].

### 3.3. Characterization of Hydration Products

#### 3.3.1. XRD Patterns

**Figure 7:**
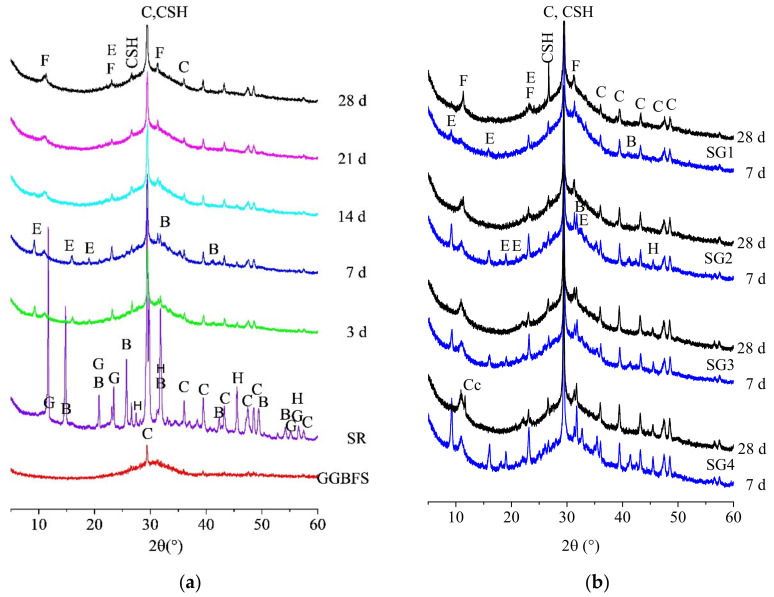
XRD patterns of (**a**) SR, GGBFS and SG2 at 3, 7, 14, 21 and 28 d; (**b**) SG1-SG4 at 7 and 28 d (B, Bassanite; CSH, Calcium Silicate Hydrates; C, Calcite; Cc-Calcium Chloride; E, Ettringite; F, Friedel’s Salt; G,Gypsum; H,halite).

XRD can judge the changes of the hydration products before and after the reaction of the pastes. The hydration products of the pastes are diverse depending on proportion of SR addition and curing time. Figure 7 shows the XRD patterns of raw materials (SR, GGBFS), SG2 at different curing times (3, 7, 14, 21, and 28 d), and SG1-SG4 at 7 and 28 d.

As Figure 7a shows, with curing time increasing from 3 to 28 d, the reflections of the hydration products of SG2 show regular changes. At 3 d, the hydration products are AFt (2θ = 9.247, 16.118, 19.116, 23.197), Fs (2θ = 10.913, 23.530, 31.441) and C–S–H gels (2θ = 26.778, 29.526). Besides, the reflections of calcite (2θ = 29.526, 39.561, 43.392, 47.598, 48.597, 57.550), basanite (2θ = 31.816, 41.301) and halite (2θ = 45.557) also can be seen. Studies [11,41,47] showed that the OH^−^ ion (released from SR) could break Al-O-Si, Si-O-Si, Al-O-Al and Ca-O bonds of GGBFS, causing GGBFS to release [SiO_4_]^4−^, [AlO_4_]^5−^, Ca^2+^, Ca(OH)^+^ and Ca(H_2_O)OH^+^ ions, which react with Ca^2+^, [SO_4_]^2−^, Cl^−^ and OH^−^ ions released from SR, and form AFt, Fs and C-S-H gels. As is shown in Figure 7a, during the whole curing time, the characteristic reflections of Fs and C–S–H gels always exist and gradually increase, and become sharper at 28 d.

It is worth noting that with the increase of curing time, the characteristic reflections of gypsum, basanite and halite gradually decrease and then disappear at 14 d, which indicates that gypsum, basanite and halite are fully consumed at this time. Additionally, characteristic reflections strength of AFt first increases and then decreases from 3 to 14 d, with its maximum at 7 d. The characteristic reflections of AFt can hardly be seen at 21 and 28 d, indicating that the amount of ettringite is low in SG2. This corresponds with a previous study [12], in which 6% gypsum was added to the alinite cement (SR is one of the raw materials of synthetic cement). He et al. [10] prepared clay samples solidified with SR and GGBFS and soaked them in NaCl (30 g/L) and NaCl (30 g/L) + MgSO_4_ (15 g/L) solution, respectively, finding that when the samples were cured or soaked for 28 d, AFt was not prominent.

As shown in Figure 7b, with the increase of SR addition, no new hydration products are generated at 7 d. The characteristic reflections of AFt, Fs and C-S-H gels become stronger. Bassanite and halite, not fully involved in the hydration reaction, still exist. At 28 d, little characteristic reflections of AFt can be found in all pastes. In comparison, basanite and halite still exist in SG3 and SG4, which mainly because with the increase of SR addition, the amount of [SO_4_]^2−^ and Cl^−^ ions in the pastes increases, and beyond the ability of the pastes to bind [SO_4_]^2−^ and Cl^−^ ions.

#### 3.3.2. FTIR Spectra

**Figure 8:**
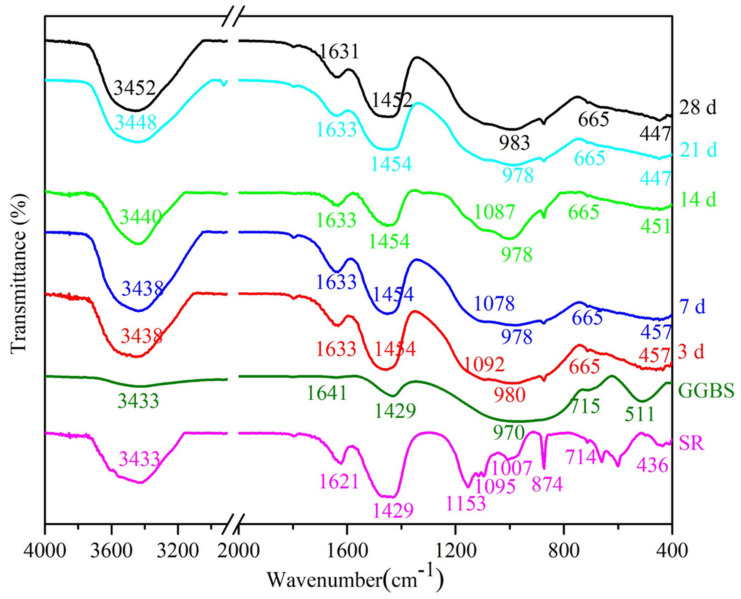
FTIR spectra of SR, GGBFS and SG2 at different curing times (3, 7, 14, 21, and 28 d).

FTIR spectra (400–4000 cm^−1^) of raw materials (SR and GGBFS) and SG2 at different curing times (3, 7, 14, 21, and 28 d) are presented in Figure 8. As shown in Figure 8, in all the tested pastes, broad bands appear around 3431–3452 cm^−1^ due to stretching vibrations of -OH and H-O-H bonds [48]. Meanwhile, the peak at 1650 cm^−1^ corresponds to the in-plane bending vibration of the H-O-H bonds [48,49]. According to the analysis of Section 2.1 and Section 3.3.1, SR contains Ca(OH)_2_, CaCl_2_⋅H_2_O, CaSO_4_⋅2H_2_O, and CaSO_4_⋅0.5H_2_O, leading to the appearance of –OH and H–O–H bonds in the SR. –OH bonds in SG2 are due to the hydration products, AFm (calcium monosulfoaluminate), AFt, Fs, and C–S–H gels. 

The O–C–O stretching vibrational bands of SG2 are found around 1429–1454 cm^−1^ [48,50] and at 874 cm^−1^ [51] at all curing times. SG2 was cured in sealed plastic bags, so [CO_3_]^2−^ was from raw materials, SR and GGBFS. 

The O-S-O asymmetric stretching vibrations bands at 1153, 1095, 660 and 600 cm^−1^ [52] can be clearly seen in SR due to CaSO_4_⋅2H_2_O and CaSO_4_⋅0.5H_2_O in SR, whereas the O-S–O bond in SG2 is almost invisible in all hydration ages (3, 7, 14, 21, and 28 d), indicating that the amount of AFt in SG2 is low at these hydration ages, which is consistent with the results of XRD analysis.

With the increase of curing time, the stretching band Si–O in GGBFS at 970 cm^−1^ moves to higher frequency among 978–983 cm^−1^, indicating that the polymerization of Si–O bond occurs. Additionally, the broad bands appearing around 457–447 cm^−1^ are due to O–Si–O bending modes of SiO_4_ tetrahedra of the GGBFS [22], indicating the existence of unreacted GGBFS in the pastes.

#### 3.3.3. SEM-EDS Analyses

**Figure 9:**
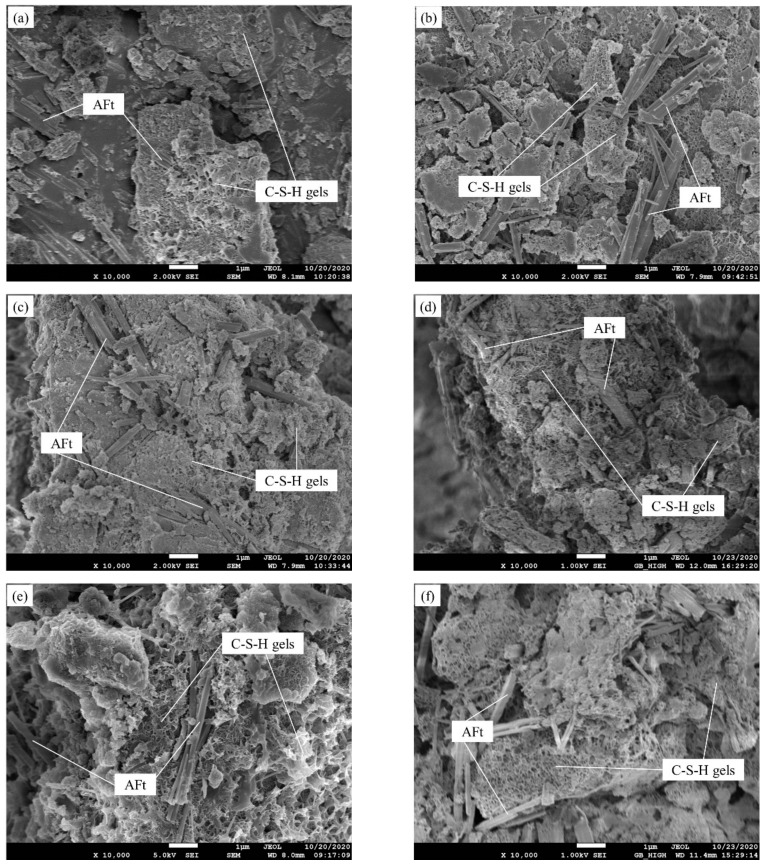

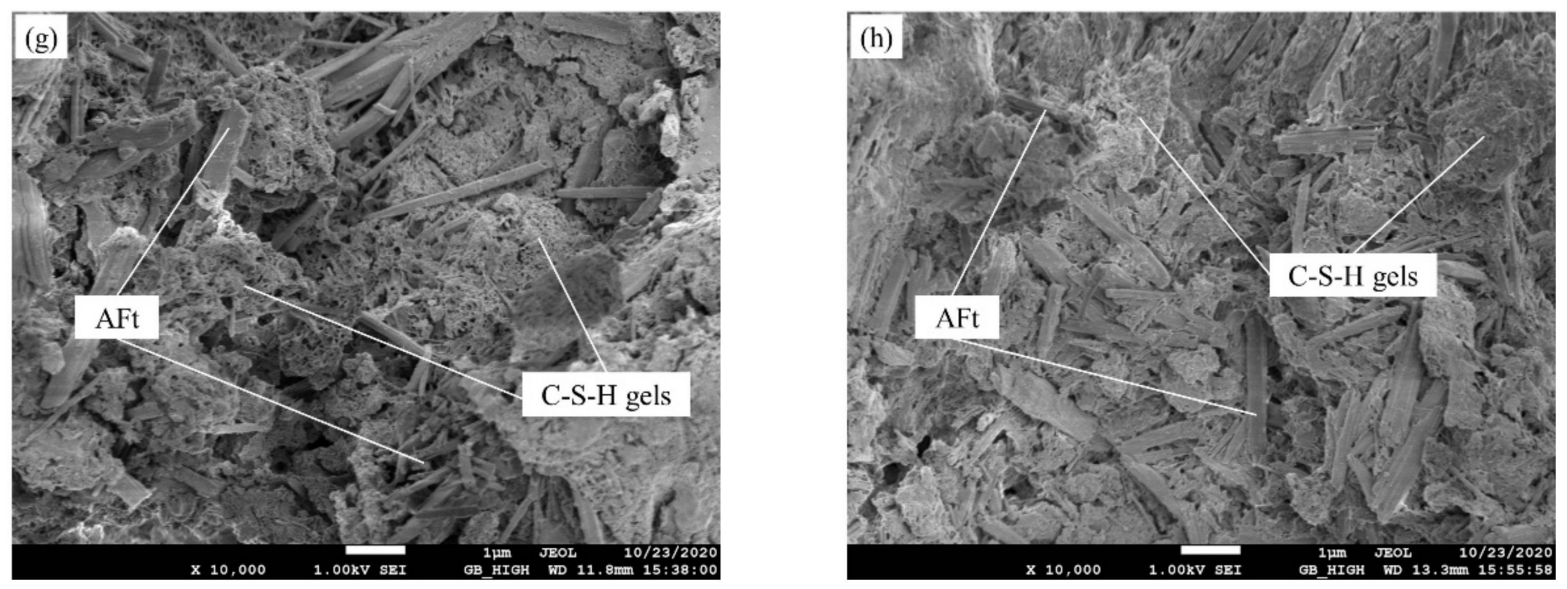
SEM images of pastes: (**a**) SG2, 3 d; (**b**) SG2, 7 d; (**c**) SG2, 14 d; (**d**) SG2, 21 d; (**e**) SG1, 28 d; (**f**) SG2, 28 d; (**g**) SG3, 28 d; (**h**) SG4, 28 d.

Figure 9 shows SEM images of SG2 at different curing time (3, 7, 14, 21 and 28 d) and of SG1-SG4, at 28 d. In SG2, the irregular GGBFS particles with clear edges are found at 3 d (Figure 9a). Small amounts of needle-like AFt and honeycomb-like C–S–H gels can be seen, and these hydration products either fill the pores or adhere to GGBFS surface. As the curing time increases to 7 d (Figure 9b), AFt increases, and lots of AFt fills the space among the particles. AFt in SG2 cured for 14, 21 and 28 d (Figure 9c,d,f) is obviously less than that in SG2 cured for 7 d, and most of it is coated with hydrated gels. The longer the curing time (from 7 to 28 d) is, the less AFt is in SG2, and the more C–S–H gels are filled in the pores and adheres to the particles. When the curing time increases to 28 d, a large amount of hydrated gels makes the structure of SG2 denser. As can be observed in Figure 9e–h, pore microstructure and the amount of AFt change with the variation of SR addition at 28 d. With the increase of the SR addition, the amount of AFt increases. This phenomenon may be attributed to the fact that with more SR addition, more CaSO_4_ exists, which increases the production of AFt. In Figure 9h (SG4), large amounts of AFt are attached to the surface of unreacted GGBFS particles to hinder the hydration of GGBFS, which may be another reason for the low strength of SG4. SEM analysis of the changes of AFt in the pastes is coincident with the XRD results (Figure 7).

**Figure 10:**
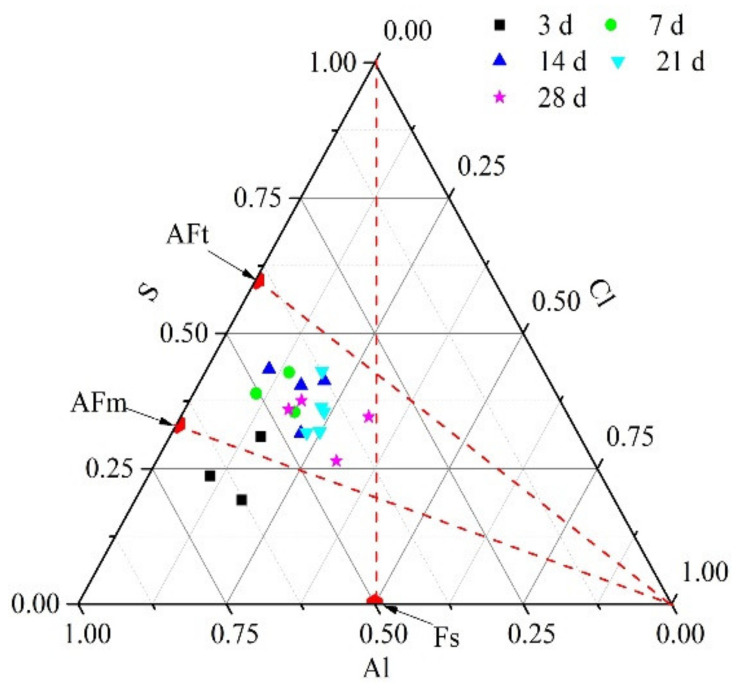
Equivalent ternary diagram of Al, S, and Cl elements in the hydration products of SG2 at 3, 7, 14, 21, and 28 d.

With the coexisting phenomenon of the hydration products, AFt, AFm, and Fs in the cementitious materials, competition coexisted between Cl^−^ and [SO_4_]^2−^ ions present in the solution [47,53,54]. The changes of the amount of ettringite in SG2 at different hydration age (3, 7, 14, 21, and 28 d) were further analyzed by SEM-EDS. EDS spots of Al, S, and Cl elements in the hydration products are shown in the Al-S-Cl equivalent ternary diagram (Figure 10). Molar ratio of Cl/Al in Fs (3CaO⋅Al_2_O_3_⋅CaCl_2_⋅10H_2_O) is 1:1. Molar ratio of S/Al in AFm (3CaO⋅Al_2_O_3_⋅CaSO_4_⋅12H_2_O) is 1:2. Molar ratio of S/Al in AFt (3CaO⋅Al_2_O_3_⋅3CaSO_4_⋅32H_2_O) is 3:2. Based on that, spots of Fs, AFm and AFt are illustrated in Figure 9. As shown in Figure 9, the amount of S element in SG2 rises to the direction of AFt from the curing time of 3 d to 7 d, whereas as the curing time continues to increase, it moves the direction of AFm. As can be seen in Figure 7a, the reflections of ettringite phases have the same phenomenon. It may because with the hydration reaction, a large number of calcium and aluminum phases lead to the transformation of AFt to AFm. It is worth noting that the amount of Cl element in SG2 rises in all curing times. It suggests that at the initial stage of hydration reaction, the hydration products of AFt, AFm and Fs coexist. As the reaction progresses, more and more calcium and aluminum phases are dissolved from GGBFS, resulting in the transformation of AFt to AFm. Cl^−^ ion in the solution further reacts with AFm to generate Fs. It is consistent with the results of XRD analysis.

### 3.4. Chloride Binding of the Pastes

Studies showed that chloride ingress and binding in concrete were significant factors in the corrosion of steel in reinforced concrete structures and elements [28]. Chloride ion was mainly found in the pore solution of concrete and the binding chloride in hydration gels [55]. Additionally, the free chloride in the pore solution is the main reason for the corrosion of steel. Therefore, free chloride ion in the SR-activated GGBFS pastes is of great significance to its application in civil engineering. It was proved that GGBFS had an excellent ability to bind chloride ion due to the high aluminate levels present in GGBFS [56].

**Figure 11:**
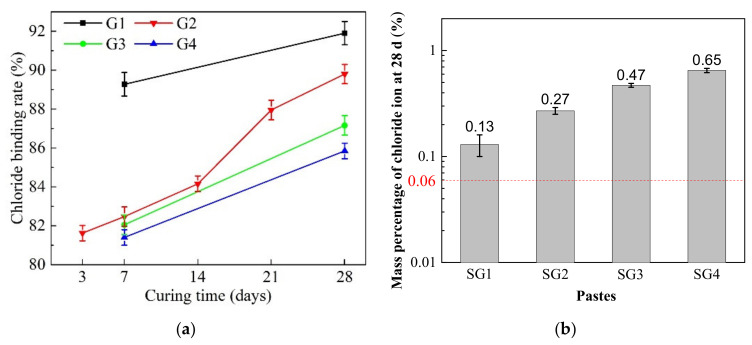
(**a**) Chloride binding rate of the pastes with different SR addition at different curing times; (**b**) Weight percentage of free chloride ion in the pastes.

Figure 11a shows the chloride binding rate of the SR-activated GGBFS pastes with different SR addition at different curing times. It can be seen that about 81.62% chloride ion has been bound in SG2 at 3 d. As the curing time increases, the chloride binding rate increases linearly from 3 to 14 d, whereas the chloride binding rate increases sharply after 14 d. At 28 d, the chloride binding rate is as high as 87.80%. It indicates that with the hydration reaction proceeding, more chloride ion is solidified by hydration products of the pastes. Chloride binding rate of SG1, SG2, SG3, and SG4 rises with the increase of curing time. At 28 d, the chloride binding rate of the pastes decreases from 91.90% (SG1) to 89.80% (SG4). According to the Chinese National Standard GB 175–2007 [57], Cl^−^ in cement should be less than 0.06%. As shown in Figure 11b, the mass percentage of free chloride ion in the pastes at 28 d reveals a rising trend with the increase of SR addition. One reason is that although the hydration reaction lasts for 28 d, free chloride ion may still exist in the SR, as discussed in Section 2.1. Another reason is that with the increases of SR addition, the amount of chloride element in the initial pastes increases, which exceeds the binding capacity of hydration products. When the proportion of SR addition is 8%, the mass percentage of free chloride ion is 0.13%, which is much higher than the requirements of the specification (blow 0.06%). Therefore, utilizing the pastes instead of cement to prepare reinforced concrete may corrode the steel. Ma, J. et al. [6] utilized SR, fly ash, lime, sand and rubble to prepare soda residue soil for geotechnical engineering, and the subgrade bearing capacity and deformation modulus of the soil in field tests were more than 210 kPa and 34.48 MPa, respectively. Zhao et al. [14] utilized SR, fly ash and sodium silicate solution to prepare fly ash-based geopolymer pastes for goaf backfill. Xu, D. et al. [58] used SR, GGBFS, steel slag, and flue gas desulfurization gypsum as cementitious materials, and waste iron ore tailings as aggregates to synthesis clinker-free concretes. They suggested that a reinforcement rust inhibitor was indispensable to avoid the steel corrosion in the concretes. Therefore, SR activated GGBFS cementitious materials can be used to replace cement in the field of civil engineering without reinforcement.

### 3.5. Hydration Mechanism

**Figure 12:**
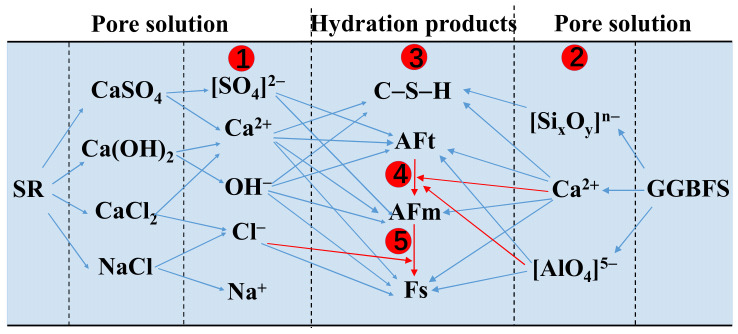
Schematic diagram of the hydration mechanism of SR-activated GGBFS pastes. The blue background represents the aqueous solution of the reaction; The symbols, ①, ②, ③, ④, and ⑤, represent the first, second, third, fourth, and fifth stage of the reaction, respectively.

Schematic diagram of the hydration mechanism of the pastes is described in Figure 12. During the first stage of hydration, a part of the SR substances dissolves in aqueous solution, releasing Na^+^, Ca^2+^, OH^−^, [SO_4_]^2−^, and Cl^−^ ions. In the second stage, under the strong action of OH^−^ ions (pH value ≈ 12), the “protective film” on the surface of GGBFS begins to be decomposed and releases calcium-rich, silica-rich, and aluminum-rich phases into the solution. In the third stage, Ca^2+^ ions in the solution combine with the silica-rich phase to form C–S–H gels, which can be expressed by the Equation (9). As the aluminum-rich phase of GGBFS is released slowly, a large amount of [SO_4_]^2−^ and Cl^−^ ions in the solution reacts with it immediately to generate AFt, as shown in Equation (2), and Fs, as shown in Equations (3) and (10), respectively. In the fourth stage, as the hydration reaction continues, GGBFS is further decomposed, more aluminum-rich phase is dissolved in the solution, and [SO_4_]^2−^ ions relatively decreases.

When the amount of sulfate is not enough to continue to synthesize AFt, AFt converts to AFm, as Equation (11) shows. At the fifth stage, free Cl^−^ ions in the solution continue to react with AFm to form Fs [47], as is shown in Equation (12). It is worth noting that when a large amount of AFt is present in the solution and adheres to the surface of GGBFS (Figure 9h), hydration reaction is hindered. Additionally, C–S–H gels can also physically adsorb [SO_4_]^2−^ and Cl^−^ ions, which will not be discussed in this study:

(9) $$\mathrm{xCaO}+{\mathrm{ySiO}}_{2}+{\mathrm{nH}}_{2}O\to \mathrm{xCaO}\cdot {\mathrm{ySiO}}_{2}\cdot {\mathrm{nH}}_{2}O$$

(10) $$2\mathrm{NaCl}+3\mathrm{CaO}\cdot {\mathrm{Al}}_{2}O_{3}\cdot 6H_{2}O+\mathrm{Ca}{\left(\mathrm{OH}\right)}_{2}+4H_{2}O\;\to 3\mathrm{CaO}\cdot {\mathrm{Al}}_{2}O_{3}\cdot {\mathrm{CaCl}}_{2}\cdot 10H_{2}O+2\mathrm{NaOH}$$

(11) $$3\mathrm{CaO}\cdot {\mathrm{Al}}_{2}O_{3}\cdot 3{\mathrm{CaSO}}_{4}\cdot 32H_{2}O+6\mathrm{CaO}+2{\mathrm{Al}}_{2}O_{3}+4H_{2}O\to \;3\left(3\mathrm{CaO}\cdot {\mathrm{Al}}_{2}O_{3}\cdot {\mathrm{CaSO}}_{4}\cdot 12H_{2}O\right)$$

(12) $$3\mathrm{CaO}\cdot {\mathrm{Al}}_{2}O_{3}\cdot {\mathrm{CaSO}}_{4}\cdot 12H_{2}O+2{\mathrm{Cl}}^{-}\to \;3\mathrm{CaO}\cdot {\mathrm{Al}}_{2}O_{3}\cdot {\mathrm{CaCl}}_{2}\cdot 10H_{2}O+{\left[{\mathrm{SO}}_{4}\right]}^{2-}+2H_{2}O$$

## 4. Conclusions

In this study, SR and GGBFS were used to prepare alkali-activated cementitious materials to provide the basis for waste recycling. Physical properties, mechanical strength, hydration products, and hydration mechanism of the SR-activated GGBFS pastes were studied. Based on detailed experimental analyses, the following meaningful conclusions can be drawn:

(1) The addition of SR was good to reduce the density of the SR-activated GGBFS pastes, whereas it increased water absorption and porosity. 24–32% SR addition led to uneven mixing of SR and GGBFS.
(2) The proper proportion of SR in the SR-activated GGBFS pastes was 16%. The higher the SR addition was, the lower the strength of the pastes was. The maximum compressive strength (34.1 MPa) and flexural strength (6.9 MPa) occurred when the proportion of SR addition was 16% at 28 d.
(3) The main hydration products of the SR-activated GGBFS pastes were C-S-H gels, AFt, and Fs. The amount of AFt in the pastes varied with the amount of SR addition and curing time.
(4) SR contains CaCl_2_, NaCl, Ca(OH)_2_, and CaSO_4_, and can act as an alkaline activator in the SR-activated GGBFS pastes. GGBFS has a high capacity for binding chloride ion in SR. With the SR addition increasing from 8% to 32%, chloride binding rate of the pastes decreased from 91.90% to 89.80% at 28 d.
(5) The percentage of free chloride ion in SR-activated GGBFS pastes is much higher than the requirements of the specification (blow 0.06%), and has the possibility of corroding steel. Therefore, SR activated GGBFS cementitious materials can be used to replace cement in the field of civil engineering without reinforcement.

The compressive strength of SR (16%)-activated GGBFS pastes can reach 34.1 MPa at 28 d, whereas the free chloride ion in the pastes may corrode steel in the concretes. In the following study, different admixtures will be added to test the pastes’ properties and ability to bind chloride ion.

## Figures and Tables

**Table 1:** Chemical compositions (wt.%) and physical properties of SR and GGBFS [5].

Components	SR	Components	GGBFS
CaCO_3_	39.6	CaO	41.4
CaCl_2_	13.4	SiO_2_	28.1
Ca(OH)_2_	11.2	Al_2_O_3_	14.8
CaSO_4_	9.8	MgO	9.5
MgO	7.0	Fe_2_O_3_	1.4
SiO_2_	6.5	TiO_2_	1.1
NaCl	6.0	Na_2_O	0.6
Al_2_O_3_	2.0	K_2_O	0.6
Others	4.5	Others	2.5
**Physical Properties**	**SR**	**-**	**GGBFS**
Color	grayish	-	white
BET surface area (m^2^/g)	409	-	450
Specific gravity	2.25	-	2.87

## Data Availability

The data presented in this study are available on request from the corresponding author. The data are not publicly available due to the privacy restrictions.
